# Robotics in uro-oncologic surgery

**DOI:** 10.3332/ecancer.2013.354

**Published:** 2013-09-26

**Authors:** Elisa De Lorenzis, Carlotta Palumbo, Gabriele Cozzi, Michele Talso, Marco Rosso, Beatrice Costa, Franco Gadda, Bernardo Rocco

**Affiliations:** Department of Specialist Surgical Sciences, University of Milan, Urology Department, Fondazione IRCCS Ca’ Granda Ospedale Maggiore Policlinico, Milan, Italy

**Keywords:** robotics, minimally invasive surgery, cystectomy, nephroureterectomy, nephrectomy, partial nephrectomy

## Abstract

In urology, the main use for the robotic technique has been in radical prostatectomy for prostate cancer. Robotic surgery for other organs, such as the kidneys and bladder, has been less explored. However, partial nephrectomy or radical nephroureterectomy can be difficult for inexperienced laparoscopic surgeons. The advent of the da Vinci robot, with multijointed endowristed instruments and stereoscopic vision, decreases the technical difficulty of intracorporeal suturing and improves the reconstructive steps.

The objective of this article is to offer an overview of all robotic procedures recently developed in the field of urology. We evaluate the feasibility of these procedures and their potential advantages and disadvantages. We also describe perioperative, postoperative, and oncologic outcomes of robot-assisted surgery as well as perform a comparison with open and laparoscopic techniques.

Comparative data and an adequate follow-up are needed to demonstrate equivalent oncologic outcomes in comparison with traditional open or laparoscopic procedures.

## Introduction

Laparoscopic surgery represented a major breakthrough in the urologic field, due to the decreased intraoperative estimated blood loss (EBL), shorter hospital stay, and quicker return of the patient to function [1]. The main obstacle that prevented the widespread use of the laparoscopic approach is the steep learning curve required for a surgeon to achieve proficiency [2].

The advent of robotic-assisted laparoscopic surgery represented a great advantage for experienced laparoscopic surgeons and for laparoscopically naïve ones.

Urology is the surgical specialty that really introduced the possibilities of robot-assisted surgery and ensured its very rapid development with its application to the radical prostatectomy procedure.

Urologists experienced in laparoscopy found in the robotic-assisted approach a better quality of vision, with 3D resolution, precise movements, and no limitations on movements. On the other hand, open surgeons were provided with a minimally invasive technique with a simpler and faster learning curve [3].

In the field of oncologic urologic surgery, the robotic approach has been applied to radical prostatectomy, radical cystoprostatectomy, partial and radical nephrectomy, and nephroureterectomy [2].

## Robot-assisted radical cystoprostatectomy

Radical cystectomy is the gold standard of treatment for patients with muscle-invasive bladder cancer (BC) and for those with high-risk recurrent noninvasive disease, in terms of local control and long-term disease-free survival [4].

In recent years, a case series has been reported in the literature of radical cystectomy performed with minimally invasive approaches such as laparoscopic and robotic-assisted techniques [5]. These reports have demonstrated the feasibility of robot-assisted radical cystectomy (RARC); the potential benefits of this procedure are lower surgical blood loss, more rapid return of bowel function, and a shorter hospitalisation [6, 7].

However, due to the lack of sufficient comparative data and long-term follow-up, there is diffidence about the real advantages of the laparoscopic and robotic approaches in comparison with traditional open radical cystectomy (ORC).

Currently, high levels of clinical evidence of the benefits of RARC are lacking in peer-reviewed literature, because the majority of the reports are single-institution case series and because randomised clinical trials are lacking or with a limited number of patients enrolled [8].

For these reasons, both RARC and laparoscopic radical cystectomy (LRC) are still considered experimental [9].

### Indications for RARC

Indications for RARC are the same as those for ORC contemplated in the European Association of Urology Guidelines: ‘localized BC (cT2N0M0) or locally advanced BC (cT3a-T4a, N0-NX, M0). Radical cystectomy may be an appropriate initial treatment for some patients with first occurrence of high-risk non muscle invasive BC or recurrent non muscle invasive BC. Patients with high-grade, non muscle invasive disease (Ta-T1) or carcinoma in situ that recurs following treatment with intravesical bacillus Calmette-Guérin or patients with intermediate-risk papillary disease that cannot be controlled with transurethral resection and intravesical therapy can be candidated to radical cystectomy’ [10].

Often patients undergoing RARC represented a highly selected population as compared with concurrent patients undergoing an open approach.

The presence of bulky lymphadenopathy, locally advanced disease (T4), uncorrected coagulopathy, and morbid obesity [body mass index (BMI) > 35] is a strong contraindication to minimally invasive radical cystectomy. In addition, patients with significant prior abdominal surgery and/or prior radiation therapy are often avoided because of concerns about prolonged laparoscopic adhesiolysis and the consequent risks of bowel injury and poor anastomotic healing [11, 12].

During RARC, ileal conduit or orthotopic bladder substitution can be performed with intracorporeal or extracorporeal techniques. Laparoscopic intracorporeal construction of urinary diversion with or without robotic assistance has been tested in small series only. It is a challenging and lengthy procedure with the current technical equipment available. This technique is considered an experimental technique, in the Guidelines of the European Association of Urology, while laparoscopic cystectomy and pelvic lymphadenectomy (with or without robotic assistance) with extracorporeal construction of urinary diversion are considered an option for surgical treatment [level of evidence (LE): 3] [10].

### Robotic-assisted lymph node dissection during RARC

During radical cystectomy, along with the bladder and the surrounding perivesical tissues and pelvic lymph nodes, the adjoining organs are also removed. In men, the prostate and the seminal vesicles are removed, while in women, the ovaries, fallopian tubes, and uterus may be removed. Thus, RARC must be able to replicate the lymphadenectomy performed with open surgery to be considered as an alternative technique [13].

The feasibility of pelvic lymph node dissection during RARC as well as the adequacy of nodal yield has been a matter of concern. However, the improved range of movement of the da Vinci S and Si systems has placated many of these preoccupations. Acceptable lymph node yields have been reported in RARC series, with mean lymph node counts up to 18 (range: 6–43) [14].

A non-randomised comparative study has reported equivalence between ORC and RARC lymph node yields (15 versus 16) [15]. Another randomised, prospective trial showed equivalence in node counts with a mean of 18 nodes harvested in the open group and19 nodes in the robotic group [8].

A prospectively maintained but retrospectively analysed study based on the International Robotic Cystectomy Consortium database concluded that the rates of lymphadenectomy at RARC for advanced BC are similar to those of ORC series [16]. The learning curve could certainly play a role in the number of nodes removed during pelvic lymph node dissection, meaning that robotic surgeons remove more nodes as their case series increase.

### Perioperative and postoperative outcomes of RARC

There is a lack of consensus in the way of reporting complications in urological surgery. For this reason, comparisons of surgical results between different techniques can be difficult [17]. With the introduction and the spread of the Clavien classification, the system of complications reporting is now defined and standardised [18].

The perioperative outcomes of RARC seemed to be comparable with ORC series [19]. A study comparing postoperative complications in 104 ORCs versus 83 RARCs has suggested a decrease in the rate of high-grade complications compared with ORC, with a 17% major complication rate in patients undergoing RARC compared with a 31% high-grade complication rate observed in ORC patients. The authors concluded that RARC is an independent predictor of fewer overall and major complications [20].

Smith *et al* [21] reviewed RARC’s technique and associated preoperative and oncological outcomes. They concluded that, although long-term data are not yet available, RARC is effective in decreasing hospital stay, analgesic requirement, time to return of bowel function, and improving health-related quality of life.

A recent prospective cohort comparison of 158 patients undergoing ORC (*n *= 52), LRC (*n *= 58), or RARC (*n *= 48) showed that RARC had the lowest transfusion and complication rates and the shortest length of stay. However, operating time was shorter in ORC and LRC compared with RARC [22].

The only prospective randomised controlled noninferiority study [8] that compared ORC versus RARC procedures demonstrated lower mean EBL (575 versus 258 mL), lower analgesic requirements (147.4 versus 89.0 mg), and earlier return to bowel function (4.3 versus 3.2 days) in favour of robotic assistance. The authors observed similar complication rates between the two procedural groups and significantly longer operating room time in the robotic group (4.20 hours) versus the open cohort (3.51 hours).

### Oncologic outcomes after RARC

Long-term oncologic outcomes to define the place of the robot-assisted approach for BC are awaited; there are concerns over the ability of minimally invasive radical cystectomy to treat bulky and locally advanced disease, making careful patient selection vital. More definitive long-term data are still needed, and ultimately head-to-head randomised comparisons will provide the most useful data [23].

In accordance with Dasgupta *et al* [24], intermediate oncological outcomes are encouraging and comparable with the contemporary ORC series. Indeed, the authors reported 100% overall and recurrence-free survival at 2 years, while at a maximum follow-up of 3.5 years, the overall and recurrence-free survival were 95% and 90%, respectively.

A recent collaborative review [23] seems to indicate that in patients with BC ≤ pT2, LRC and RARC achieve comparable, acceptable positive surgical margins (PSMs) to ORC. However, in those with higher-stage disease (pT4) and perhaps in those with pT3 disease, PSM rates are higher, and the loss of tactile feedback or inexperience with the technique may contribute to this rise.

## Robotic-assisted radical nephrectomy

Open radical nephrectomy (ORN) is the gold standard of treatment for large renal tumours and has demonstrated good long-term oncologic outcomes. Since the first laparoscopic nephrectomy in 1991 [25], the use of a minimally invasive approach has become the preferred treatment modality for most nephrectomy cases.

The potential benefits of the da Vinci surgery can be more evident during reconstructive rather than during ablative procedures. For this reason, there is less appeal to use this technology for the ablative surgery of the upper urinary tract (UUT) [26–28].

Potential advantages of robotic assistance for performing a radical nephrectomy are a magnified 3D high-definition vision and the movements of the EndoWrist instruments that can facilitate a careful dissection and ligation of the renal hilum. Nevertheless, robotic-assisted radical nephrectomy (RARN) can be used as training during the learning curve for acquiring the ability needed for more complex types of kidney surgery, such as a partial nephrectomy [29]. However, RARN has certain definite disadvantages in the form of high cost, the set-up time, need for training in its use, lack of tactile feedback, and the need for an experienced laparoscopic assistant [30].

### Indications for RARN

The principles for RARN are primarily the same as those for laparoscopic radical nephrectomy (LRN). In the Guidelines of the European Association of Urology, LRN is considered the standard of care for patients with T2 tumours and smaller renal masses not treatable by nephron-sparing surgery (NSS) [31].

Few reports discuss robotic nephrectomy, and those that do are based on small patient cohorts [32].

### Perioperative, postoperative, and oncological outcomes after RARN

The surgical approach for robotic nephrectomy included both transperitoneal and retroperitoneal approaches.

RARN for renal cancer has been reported in the literature in very few published series that demonstrated the feasibility and viability of RARN, as an alternative for performing radical nephrectomy [27, 32].

Nazemi *et al* [30] compared radical nephrectomy performed with open, laparoscopic, hand-assisted laparoscopic, or robotic-assisted approach. No differences in terms of perioperative complications were detected between the methods. While EBL, postoperative narcotic doses, and hospitalisation were significantly higher in the open group, the median operative time was significantly longer for RARN.

Only one small, non-randomised prospective cohort study [33] compared RARN with LRN. The groups were similar at baseline in terms of tumour size, stage, and grade, histological cell type, and age, but the study provided no baseline data on necrosis, ethnicity, performance status, and co-morbidity and was therefore assessed at high risk of confounding from these factors. Follow-up was short. There was no clear difference between the groups in terms of EBL, need for blood transfusion, analgesic requirements, hospital stay, and convalescence time. The mean operating time was significantly longer with the use of the robot. Both RARN and LLR cases had no recurrence or metastasis. No survival analysis was reported. The authors concluded that in their comparative study, there were no extraordinary benefits of RARN observed over LRN for localised renal tumour.

Murphy and Dasgupta [26] concluded that in their article about robotic approaches to renal cancer the small number of reported cases managed with RARN suggests that this procedure is unlikely to enter standard practice.

## Robotic-assisted partial nephrectomy

Surgical therapy is the only curative therapeutic approach for the treatment of renal cell cancer. For T1 tumours, radical nephrectomy is no longer the gold standard treatment [31].

Robot-assisted partial nephrectomy (RAPN) allows magnified stereoscopic visualisation and the use of articulated robotic instruments under precise control [34]. The advent of robot assistance has facilitated the technical performance of partial nephrectomies by simplifying and speeding up two important parts of the procedure: dissection of the renal parenchyma and suturing after tumour resection. The use of a robot facilitates the dissection and mobilisation of the kidney, especially for the approach to the upper pole, which is sometimes difficult in standard laparoscopy.

Since its introduction by Gettman *et al* in 2004 [35], there has been a steady increase in RAPN, with more than 300 cases reported between 2009 and 2010 [36–39]. It is becoming the technique of choice for most stage T1a tumours, where the technology and expertise are available.

### Indications for RARN

As indicated in the European Association of Urology Guidelines, for elective indications, NSS (partial tumour resection) for tumours limited in diameter (T1a) provides recurrence-free and long-term survival rates similar to those observed after radical surgery (LE: 2b). For larger tumours (T1b), partial nephrectomy has demonstrated feasibility and oncological safety in carefully selected patients [31].

NSS is recommended when a small renal mass (defined as a ‘contrast-enhancing mass within the kidney with the largest dimension ≤4 cm’ [40]) was detected in anatomically or functionally solitary kidneys (absolute indications), in patients with a contralateral functioning kidney with a condition that might impair renal function in the future, or in the presence of multiple bilateral tumours or hereditary forms of renal cell cancer that represent a high risk of developing a tumour in the contralateral kidney (relative indications) [41]. In the last few years, the evidence derived from high-quality clinical research and randomised trials has increased the use of partial nephrectomy in the presence of localised unilateral renal cell cancer with a healthy contralateral kidney (elective indications) [34].

However, in the European Association of Urology Guidelines on Renal Cell Carcinoma, RAPN is considered a novel technique that is still undergoing evaluation [31].

### Perioperative and postoperative outcomes of RAPN

Overall, RALPN seemed to afford significant improvements in operative time, warm ischaemia time (WIT), and EBL. These improvements have been demonstrated by several authors [39, 42–46].

One small, non-randomised study [47] conducted a matched-pair study on the basis of age, gender, BMI, ASA score, tumour size, location, and specific technique used (early versus conventional unclamping) in 12 patients who underwent robotic and in 12 who had standard LPN. Perioperative outcomes were comparable for operative blood loss, operation time, WIT, and length of hospital stay. Two patients in the RAPN group were converted to standard LPN, one for a positive focal margin on frozen-section analysis, and the other for a robotic camera malfunction. The renal functional outcomes as measured by 3-month serum creatinine and estimated GFR were comparable between the matched groups.

The first large, comparative, multi-institutional study was reported by Benway *et al* in 2009 [42]. They evaluated the outcomes of three experienced minimally invasive surgeons by analysing 118 consecutive LPNs and 129 consecutive RAPNs. No significant differences were detected in overall operative time or PSM rate. They also found that increased tumour complexity resulted in increased LPN operative time. This was not the case for RAPN, perhaps indicating that the ease of tumour dissection and repair allows for a more uniform experience with RAPN than for LPN. These findings support the idea that the learning curve can be shortened using robotics.

A very recent comparative analysis of a large series of laparoscopic and robotic partial nephrectomies performed by a high volume single surgeon at a tertiary care institution [48] showed that the RAPN group had lower operative time, WIT, rates of intraoperative complications, and conversion. The RAPN group also showed a lower rate of postoperative complications with a lower rate of high grade (Clavien III–V) complications.

Pierorazio *et al* [43] in their comparison of outcomes and evaluation of learning curve of 48 RAPNs and 102 LPNs concluded that progress in perioperative outcomes in RAPN is due to the improved visualisation and excision of tumour and to the following reconstruction facilitated by the endowristed robotic instruments.

Actually, WIT is a matter of discussion in the literature because the reduction of the ischaemia time can preserve the global renal function and subsequently avoid renal injury, chronic renal disease, and the related morbidity [49, 50]. Thus, RALPN may be a means to reduce WIT further and protect long-term renal function in patients in whom NSS can be safely performed.

### Oncologic outcomes of RAPN

It has been demonstrated that LPN can guarantee equivalent intermediate and long-term oncologic outcomes (in terms of surgical margin status and local recurrence) in comparison with OPN [51]. In large single-institution series [42], PSM rate was 3.9%, which is consistent with historical rates for OPN (1.3%) and LPN (2.9%) in experienced hands [52].

In an analysis of 40 RAPN and 62 LPN cases, no significant difference was found in the rate of focal positive margins between the two modalities [39].

A recent retrospective analysis of 500 consecutive LPNs and RAPNs performed by a single surgeon showed that LPN cases had significantly more positive margins than RAPN cases overall (5.6% versus 2.9%, *p* < 0.001) [48].

The longest duration of median follow-up in the reported RAPN literature is less than 24 months [53], which is inadequate to state that oncologic efficacy. However, in this study, none of the patients had evidence of local recurrence or metastatic disease at median follow-up of 22 months.

Although the first experiences of RAPN are encouraging, oncologic outcomes are still immature, and a larger series with longer follow-up are awaited to confirm the preliminary results [34].

## Robotic-assisted nephroureterectomy

Open radical nephroureterectomy (ORNU) with excision of the ipsilateral bladder cuff has long been considered the standard management for upper urinary tract urothelial cell carcinomas (UUT-UCCs) [54].

Multiple minimally invasive techniques have been introduced with the goal of replicating these open procedures.

Robot-assisted laparoscopic nephroureterectomy (RALNU) is a relatively new procedure with promising early results. Being essentially a laparoscopic procedure, it is a minimally invasive surgery that may minimise surgical morbidity for the patient. In addition, the da Vinci surgical robot provides some additional advantage over a standard laparoscopic nephroureterectomy, namely increased degrees of freedom, 3D vision, movement scaling, and tremor filtration, and as such, it has the potential for a more widespread adoption by urologists.

### Indications for RALNU

Indications for RALNU are the same as those for ORNU: ORNU with excision of the bladder cuff is the gold standard treatment for UUT-UCCs, regardless of the location of the tumour in the UUT (LE: 3), especially for muscle-invasive and/or high-grade disease. Resection of the distal ureter and its orifice is performed because it is a part of the urinary tract with considerable risk of recurrence [54].

In the European Association of Urology Guidelines on Upper Urinary Tract Urothelial Cell Carcinomas, however, the use of robotic approach for nephroureterectomy is not mentioned [54].

### Perioperative, postoperative, and oncological outcomes after RALNU

The feasibility of RALNU by the retroperitoneal route was first described by Rose *et al* in 2006 [55].

Nanigian *et al* [56] described their experience in a series of ten patients undergoing RALNU for UUT-UCCs. A transperitoneal approach was used. No surgical complications were reported. They showed promising short-term oncologic outcomes.

Eandi *et al* [57] reported their initial experience with 11 patients who underwent to complete robotic-assisted nephroureterectomy and showed promising short intraoperative and postoperative outcomes with regard to oncological outcomes, hospital stay, and operative time. In these cases, the robotic approach required undocking and redocking of the da Vinci system during the procedure in order to reach the bladder for the excision of the bladder cuff.

Later, other authors described several robotic approaches in which the repositioning of the patient from flank to supine and movement of the patient cart are unnecessary: keeping the patient in the flank position not only shortens the operative duration but also improves exposure of the distal ureter and closure of the bladder cuff [56, 59].

More recently, Hemal *et al* [59] described their new technique in which ports were strategically placed to allow access to the kidney, ureter, and bladder: as described the excision of the bladder cuff does not require undocking the robot without the need to reposition the patient.

Despite the documented benefits of minimally invasive RALNU in perioperative morbidity, the oncological equivalence of this procedure to ORNU remains to be completely established even if overall, in the last decade, numerous retrospective studies have demonstrated oncological equivalence for laparoscopic and open nephroureterectomies [60, 61].

Short-term oncologic outcomes of RALNU have been encouraging, and it seems reasonable to expect long-term outcomes as good as those seen with traditional laparoscopy, which in turn has been comparable with the outcomes of open surgery.

Murphy *et al* [26] concluded in his article about robotic approaches to renal cancer that RALNU is technically feasible, but there are no prospective comparisons with the conventional laparoscopic approach. Randomised trials with long-term follow-up comparing the outcomes of open and robotic nephroureterectomies are needed.

## Conclusion

Advances in minimally invasive surgery have allowed urologists to use robotic assistance in developing new techniques in extirpative and reconstructive surgery.

The relatively short learning curve of robotic surgery could represent a potential advantage over laparoscopic techniques and could make robotics the minimally invasive modality of choice.

Long-term follow-up of procedures such as RARC, RAPN, and RALNU are obviously needed, but the short and intermediate parameters of efficacy of these procedures are encouraging and can predict the increased dissemination and utilisation of robotics in uro-oncologic surgery (Table 1).

## Figures and Tables

**TABLE 1.** Oncologic results in comparison with the open/laparoscopic procedure.

PROCEDURE	ONCOLOGIC RESULTS		
RARC^*^	equivalent lymph node yields	comparable overall and recurrence-free survival	similar positive surgical margins in ≤ T2 disease
RAPN^§^	comparable margin status	equivalent local recurrence rates	
RALNU^#^	studies with long-term follow-up are needed		

* robotic-assisted radical cystectomy;

§ robotic-assisted partial nephrectomy;

# robotic-assisted nephroureterectomy
